# An Exploratory Study of the Nutritional Composition and Caco-2 Safety Assessment of Elche Date Flour and Its Green Hydroethanolic Extracts

**DOI:** 10.3390/foods14223908

**Published:** 2025-11-15

**Authors:** Katarzyna Dawidowicz, Sergio Martinez-Terol, Estrella Sayas-Barberá, José Ángel Pérez-Álvarez, Francisco J. Marti-Quijal, Patricia Roig, Juan Manuel Castagnini

**Affiliations:** 1 Research Group in Innovative Technologies for Sustainable Food (ALISOST), Department of Preventive Medicine and Public Health, Food Science, Toxicology and Forensic Medicine, Faculty of Pharmacy and Food Science, Universitat de València, Avda. Vicent Andrés Estellés, s/n, 46100 Burjassot, Valencia, Spain; katarzyna2.dawidowicz@uv.es (K.D.); sergio.martinez-terol@uv.es (S.M.-T.); francisco.j.marti@uv.es (F.J.M.-Q.); patricia.roig@uv.es (P.R.); 2 IPOA Research Group, Centro de Investigación e Innovación Agroalimentaria y Agroambiental, Universidad Miguel Hernández (CIAGRO-UMH), Carretera, Beniel Km 3.2, 033121 Orihuela, Alicante, Spain; estrella.sayas@umh.es (E.S.-B.); ja.perez@umh.es (J.Á.P.-Á.)

**Keywords:** date palm fruit, carbohydrates, antioxidant activity, mineral profile, heavy metals, cell viability, valorization

## Abstract

The Elche palm grove (Spain) produces large surpluses of fresh date fruits due to low industrial processing and strict market standards. This exploratory study assessed the potential of these fruits as sustainable ingredients through the production of freeze-dried date flour and its green hydroethanolic extracts. Computer vision analysis of nine local cultivars (D1–D9) revealed broad chromatic and phenotypic diversity. Mineral and heavy metal analyses in the flour indicated high nutritional value and overall safety: D8 was richest in Mg (1.23 mg/g), P (0.78 mg/g), Fe (15.32 mg/kg), Zn (9.20 mg/kg), Cu (5.22 mg/kg), and Se (68 µg/kg), while D4 showed the highest K (22.1 mg/g) and D1 the highest Ca (1.94 mg/g). Lead and cadmium were highest in D8 and arsenic in D1, although all values remained within the regulatory limits. Hydroethanolic extracts exhibited remarkable compositional variability: D4 and D5 had the greatest carbohydrates (737.70 ± 55.79 mg/g DM), D8 and D9 the highest proteins (up to 40.31 ± 1.33 mg/g DM), and D2 and D8 the highest carotenoids (up to 36.44 ± 1.55 μg/g DM). D8 also showed the highest phenolics (13.98 ± 2.93 mg GAE/g DM) and antioxidant capacity. Cytotoxicity assays in Caco-2 cells showed no significant effects up to 1000 µg/mL. These preliminary findings suggest that green-extracted date fractions may combine nutritional richness, antioxidant potential, and biological safety, providing a basis for future studies on their application as natural and sustainable food ingredients.

## 1. Introduction

The accelerating impact of climate change poses significant challenges to the food industry and its related sectors, particularly in adopting environmentally friendly and sustainable practices. In this context, sustainable food production is critical to mitigating environmental impacts, such as reducing greenhouse gas emissions and conserving water and energy resources. Special attention has been directed toward reducing food waste and overproduction through the valorization of underutilized co-products within the Circular Economy framework [1,2].

Functional foods, designed to provide additional health benefits beyond basic nutrition, have emerged as an industrial strategy for enhancing sustainability in food systems. They often utilize co-products from food production, transforming waste into nutrient-dense products with bioactive properties. For instance, fruit and vegetable by-products have been studied extensively as sources of functional food ingredients, contributing to reduced waste and improved human health [3,4]. Moreover, functional foods play a vital role in promoting global food security by addressing malnutrition and chronic disease prevention [5].

The date palm (*Phoenix dactylifera* L.), traditionally cultivated in arid and semi-arid regions, is increasingly recognized for its nutritional richness and potential as a sustainable functional food source. While global production has expanded to new areas, such as southern Europe, limited industrial utilization and strict market specifications have resulted in fruit surpluses and postharvest losses, with up to 30% of harvested dates being discarded for not meeting commercial quality standards [6,7]. A particularly relevant example is the Palmeral of Elche (Spain), the northernmost date palm grove in the Northern Hemisphere and a UNESCO World Heritage site. This unique ecosystem produces more than 1000 tonnes of fresh dates annually, which differ from the dry dates typically consumed in North Africa and the Middle East. However, strict quality regulations, such as the recently approved Orden 11/2023 of the Valencian Regional Government, establishing the quality requirements for “Dátil de Elche” under the CV quality mark, exclude many fresh dates from the commercial market despite being suitable for consumption. As a result, only 0.02% of production is industrially processed, making dates from Elche one of the most undervalued and underutilized fruits in Spain and the European Union [7,8]. This underexploitation highlights the urgent need to explore novel strategies for their valorization as functional food ingredients.

Nutritionally, dates are an excellent source of energy, owing to their high carbohydrate content, which includes monosaccharides (glucose and fructose) and disaccharides (sucrose) [9]. They also provide significant dietary fiber, crucial for digestive health, and bioactive compounds such as phenolics, carotenoids, and flavonoids, which exhibit antioxidant, anti-inflammatory, and antimicrobial properties [10,11,12]. Dates are further rich in essential minerals (potassium, magnesium, copper, and iron) and B vitamins, making them a versatile ingredient for functional food development [7].

To date, no comprehensive study has simultaneously characterized the nutritional, mineral, and bioactive composition of Elche date cultivars, including their phenolic and carotenoid profiles, antioxidant potential, and in vitro safety assessment. This study aims to explore the nutritional and bioactive composition of date (*Phoenix dactylifera* L.) extracts derived from nine selected local date cultivars from the Elche palm grove, emphasizing its potential as a functional ingredient for the food industry. By analyzing key components such as carbohydrates, proteins, phenolic compounds, carotenoids, and minerals, alongside evaluating antioxidant capacity and bioactivity in Caco-2 cell lines, this research highlights the potential of date flour as a sustainable, health-oriented food product.

## 2. Materials and Methods

### 2.1. Plant Materials

Selected fresh dates from nine local date palm trees (*Phoenix dactylifera* L.), located in the Elche palm grove (Elche, Alicante, Spain), were manually harvested at the Tamar stage by specialized staff between September and November 2023. To facilitate comparative analysis, the samples were labeled D1 to D9. These codes represent individual trees based on the phenotypic variability, rather than commercial cultivar names. To reflect the intrinsic genetic heterogeneity of the grove, each tree was treated as an independent biological unit rather than as a replicate of a specific cultivar. After collection, dates were transported under frozen conditions and stored at −28 °C until their use at the ALISOST laboratory in the Faculty of Pharmacy and Food Science, Universitat de València (Spain).

For sample preparation, date fruits were initially frozen at −80 °C and subsequently freeze-dried for 72 h. Afterwards, the fruits were pitted, cut into smaller pieces and finally blade-milled for 2 min with a blender (Pulverisette 11, Fritsch GmbH, Idar-Oberstein,Germany). The final moisture content for the date palm flours was on average 10.8 ± 1.5% (oven drying method at 121 °C).

### 2.2. Reagents

ABTS (2,2′-azino-bis(3-ethylbenzothiazoline-6-sulfonic acid)), Trolox (6-hydroxy-2,5,7,8-tetramethylchroman-2-carboxylic acid), gallic acid (C_7_H_6_O_5_), Folin–Ciocalteu reagent and potassium persulfate (K_2_S_2_O_8_), glucose (C_6_H_12_O_6_), fluorescein (C_20_H_12_O_5_), sodium hydrogen phosphate (Na_2_HPO_4_) and potassium dihydrogen phosphate (KH_2_PO_4_) and AAPH (2,2-azobis(2-amidinopropane) dihydrochloride) were purchased from Sigma-Aldrich (Steinheim, Baden-Württemberg, Germany). Sulfuric acid (H_2_SO_4_), phenol (C_6_H_6_O), sodium carbonate (Na_2_CO_3_) and dimethyl sulfoxide (DMSO) were obtained from Thermo Fisher Scientific (Waltham, MA, USA). Ethanol (99%) was purchased from VWR Chemicals (Rosny-sous-Bois, France). DMEM, penicillin, streptomycin, trypsin/EDTA solutions, phosphate-buffered saline (PBS), fetal bovine serum (FBS) and tetrazolium bromide (MTT) were purchased from Sigma Chemical Co. (St. Louis, MO, USA).

### 2.3. Computer Vision System (CVS)

The color of the fresh samples was analyzed using a Computer Vision System (CVS) designed to ensure consistency and reproducibility in image acquisition and processing. The system consisted of a controlled illumination source, a digital camera, and custom image processing software developed in Python v. 3.10 (Python Software Foundation, USA).

Image Acquisition:

Fresh date samples were placed inside a light box constructed to exclude external light and minimize reflections, ensuring a uniform background and controlled conditions. Images were captured using a digital camera (iPhone 14 Pro, Apple Inc., Cupertino, CA, USA; 48 MP sensor) positioned vertically above the sample at a fixed distance of 35 cm. The camera was connected to a personal computer for image transfer and analysis. The lighting system comprised a daylight LED source with a D65 illuminant standard and a correlated color temperature of 6500 K, providing consistent and accurate illumination. For each sample, five replicate images were acquired under identical conditions to ensure reproducibility and minimize random variation.

Image Processing and Analysis:

Images acquired with the iPhone 14 Pro were processed using a Python script (available at: https://github.com/juancastagnini/datil-segmentation-lab, accessed on 14 November 2025). The script utilized open-source Python libraries such as scikit-image, pandas, and matplotlib to perform the following steps:

Segmentation:

A segmentation mask was created using a reference image of the samples on a white background. This mask was applied to images captured with a black background to isolate the date samples. The segmented regions corresponding to the date samples were analyzed in the CIE L*a*b* color space, ensuring an accurate representation of the sample colors independent of lighting variations. White balance was standardized using the white background inside the light box before image capture.

This method provides a robust and reproducible approach to quantify the color attributes of date samples under controlled lighting and imaging conditions.

In addition to quantitative color analysis, the acquired images allowed the visual assessment of morphological diversity among samples, supporting the qualitative observation of differences in size and shape associated with each palm.

### 2.4. Mineral and Heavy Metals Content

This study assessed the concentrations of eight essential minerals—magnesium (Mg), phosphorus (P), potassium (K), calcium (Ca), iron (Fe), copper (Cu), zinc (Zn), and selenium (Se)—as well as four heavy metals—arsenic (As), mercury (Hg), cadmium (Cd), and lead (Pb)—in date palm fruit freeze-dried samples. The essential minerals are vital for various physiological functions, including metabolic processes, tissue structure formation, and hormone synthesis. Conversely, heavy metals are known for their toxicity, making their quantification crucial for evaluating potential health risks.

The analysis was conducted using Inductively Coupled Plasma Mass Spectrometry (ICP-MS, Agilent 7900; Agilent Technologies, Santa Clara, CA, USA). Following the methodology outlined by Sebastià et al. [13], approximately 0.3 g of ground date freeze-dried fruit samples were subjected to acid digestion with 4 mL of 64% nitric acid (HNO_3_) and 1 mL of 30% hydrogen peroxide (H_2_O_2_). This digestion process, performed in a microwave system (MARS, CEM, Vertex, Spain) at 800 W and 180 °C for 15 min, effectively dissolved the organic matrix and released the target elements. Post-digestion, the samples were diluted with deionized water to the appropriate volume and introduced into the ICP-MS detector for quantification of both minerals and heavy metals. The instrument was configured with a Micromist concentric nebulizer and a Scott-type spray chamber, together with platinum interface cones, an off-axis ion lens system, a hyperbolic quadrupole mass analyzer, and an octopole collision/reaction cell operated with helium and hydrogen as collision gases.

For mineral quantification, multi-element certified reference standards of Mg, Ca, P, Fe, and Zn (10,000 μg/mL; HPS, ZeptoMetrix, North Charleston, SC, USA) were employed, using scandium and germanium (20 μg/g; ISC Science, Gijón, Spain) as internal standards. In the case of heavy metals, calibration standards for As, Cd, Hg, and Pb (10,000 μg/mL) were prepared, and a mixed internal standard solution containing Ge, Rh, and Ir (20 μg/g) was used for signal normalization. The value of the correlation coefficient was R ≥ 0.9999, and each calibration point had an RSD value of ≤5%. At the end of the sample sequence analysis, a calibration pattern was analyzed, obtaining an average between the reference and the obtained value of around RSD ≤ 5%.

This analysis was performed directly on the freeze-dried date flour instead of the hydroethanolic extract to ensure complete recovery of both bound and free elements.

All ICP-MS analyses were carried out at the Central Research Services of the Universitat de València (Servicio Central de Soporte a la Investigación Experimental (SCSIE)), an accredited facility operating under standardized QA/QC procedures including routine blanks and certified reference materials.

### 2.5. Extraction Procedure

The extracts in this study were prepared using single maceration. A 50:50 (*v*/*v*) mixture of ethanol (99%) and distilled water was used as the extraction solvent. For each extraction, 2 g of date palm flour were mixed with 10 mL of the solvent (solid-to-liquid ratio 1:5 *w*/*v*) and stirred magnetically for 30 min at 300 rpm at room temperature (25 °C) in the dark. The resulting mixtures were then centrifuged at 3000 rpm for 10 min and filtered using paper filters. For each homogenized sample, three independent hydroalcoholic extractions were performed under identical conditions to ensure reproducibility and to obtain representative extracts for subsequent analyses.

The hydroethanolic maceration applied in this work represents a simple, low-energy, and solvent-efficient green extraction method, suitable for obtaining food-grade fractions rich in bioactive compounds. The resulting extracts were subsequently used for the determination of nutritional and antioxidant properties and were also conceived as potential functional ingredients for the development of novel, sustainable food formulations.

### 2.6. Chemical Analysis

#### 2.6.1. Total Carbohydrate Content

The total carbohydrate content was determined using a colorimetric method proposed by Fournier [14]. This method involves the reaction of carbohydrates with concentrated sulfuric acid, leading to the formation of furfural and hydroxymethylfurfural, which condense with phenol to produce yellow-orange compounds. The intensity of the color is proportional to the carbohydrate content, enabling the determination of their total concentration in the analyzed extracts.

A 500 mg/L D-glucose solution was used as the calibration standard. Standards were prepared by diluting the stock solution to achieve sugar concentrations ranging from 10 to 50 mg/L. For the analysis, 1 mL of the date extract or standard was mixed with 500 μL of 4% phenol and 2.5 mL of 96% sulfuric acid. The absorbance of the reaction mixture was measured in a glass cuvette at a wavelength of 490 nm using a Perkin-Elmer UV/Vis Lambda 2 spectrophotometer (Perkin-Elmer, Waltham, MA, USA), and the results were expressed as mg of glucose equivalent per g of dry mass (mg GE/g DM).

#### 2.6.2. Total Protein Content

The protein content was determined using the Dumas method, which involves the combustion of the sample at high temperatures in the presence of oxygen, leading to the release of nitrogen gas. The released nitrogen is then measured, and its amount is used to calculate the protein content by applying a conversion factor, typically 6.25 [15,16]. In this study, ground date fruit samples were analyzed using a Thermo Scientific FlashSmart Elemental Analyzer (Thermo Fisher Scientific, Waltham, MA, USA). The results were expressed in micrograms of protein per gram of dry mass (DM).

#### 2.6.3. Total Phenolic Content

The total phenolic content (TPC) was determined using the Folin–Ciocalteu method, which relies on the redox reaction between phenolic compounds and the Folin–Ciocalteu reagent [17]. This reagent, composed of phosphomolybdic and phosphotungstic acids, reacts with phenolic substances, leading to the formation of blue-colored complexes. The intensity of this blue coloration is directly proportional to the concentration of phenolic groups present in the sample. To perform the assay, 10 μL of the sample or gallic acid standard was combined with 270 μL of 4% (*w*/*v*) sodium carbonate (Na_2_CO_3_) in a microplate. After a 5 min incubation, 10 μL of Folin–Ciocalteu reagent (50% (*v*/*v*) in distilled water) was added. The mixture was then incubated in darkness at room temperature for one hour. Subsequently, absorbance was measured at 765 nm using the BMG FLUOstar Omega microplate reader (BMG Labtech GmbH, Ortenberg, Germany). The TPC was quantified by comparing the absorbance values to a standard curve generated with known concentrations of gallic acid, and results were expressed as milligrams of gallic acid equivalents per g of dry mass (mg GAE/g DM). All measurements were conducted in triplicate to ensure accuracy and reproducibility.

#### 2.6.4. Total Antioxidant Capacity

The Total Antioxidant Capacity (TAC) of date palm extracts was assessed using two assays: Trolox Equivalent Antioxidant Capacity (TEAC) and Oxygen Radical Absorbance Capacity (ORAC).

The TEAC assay measures the ability of antioxidants to neutralize the ABTS^+^ radical cation. Following the method described by Liu et al. [18], the assay was adapted to a 96-well microplate format. A stock solution was prepared by combining 25 mL of 7 mM ABTS with 440 μL of 140 mM potassium persulfate (K_2_S_2_O_8_). This mixture was kept in the dark at room temperature overnight to generate the ABTS^+^ radical cation. The resulting solution was then diluted with 99% ethanol to achieve an absorbance of 0.70 ± 0.02 at 734 nm, establishing the initial absorbance (A_0_). In a microplate, 300 μL of this ABTS^+^ solution was mixed with 20 μL of either the sample extract or Trolox standards (an eight-point calibration curve ranging from 0 to 400 μM) and incubated in the dark for 10 min. The final absorbance (A_x_) was measured at 734 nm and 30 °C using a BMG FLUOstar Omega microplate reader (BMG Labtech GmbH, Ortenberg, Germany). Results were expressed as μmol Trolox equivalents per g of dry mass (μmol TE/g DM).

The ORAC assay evaluates the capacity of antioxidants to scavenge peroxyl radicals, thereby protecting fluorescein from oxidative degradation. The analysis was performed according to the method described by Salgado-Ramos et al. [19]. Fluorescein served as the fluorescent probe, and AAPH (2,2′-azobis(2-amidino-propane) dihydrochloride) was the peroxyl radical generator. Trolox (100 μM) was used as the standard, and phosphate buffer (pH 7.4) served as the blank. In each well of a microplate, 50 μL of fluorescein was combined with 50 μL of either the sample extract, Trolox standard, or blank, and incubated at 37 °C for 10 min. Subsequently, 50 μL of AAPH was added to initiate the reaction. Fluorescence readings were taken every minute for 95 min at excitation and emission wavelengths of 485 nm and 520 nm, respectively, using a BMG FLUOstar Omega microplate reader (BMG Labtech GmbH, Ortenberg, Germany). The antioxidant capacity was determined by calculating the area under the fluorescence decay curve (AUC) for each sample and comparing it to the AUC of the standard. Results were expressed as μmol Trolox equivalents per g of dry mass (μmol TE/g DM).

#### 2.6.5. Total Carotenoid Content

The total carotenoid content (C_x+c_) was determined spectrophotometrically following the method described by Lichtenthaler et al. [20]. Absorbance measurements were taken at 665 nm, 649 nm, and 470 nm using a Perkin-Elmer UV/Vis Lambda 2 spectrophotometer (Perkin-Elmer, Waltham, MA, USA), corresponding to the maximum absorbance wavelengths for the compounds analyzed.

EtOH 96% equations:

(1) C_a_ (μg/mL) = 13.95 A_665_ − 6.88 A_649_,

(2) C_b_ (μg/mL) = 24.96 A_649_ − 7.32 A_665_

(3) C_x+c_ (μg/mL) = (1000 A_470_ − 2.05 C_a_ − 114.8 C_b_)/245

#### 2.6.6. Cell Culture and Viability Assessment

Caco-2 Cells Culture

Human colorectal adenocarcinoma Caco-2 (ATCC HTB-37) cells were cultured in Dulbecco’s Modified Eagle’s Medium (DMEM) supplemented with 10% fetal bovine serum (FBS), 1% nonessential amino acids, 1% HEPES buffer solution, 0.1% fungizone, 100 U/mL penicillin, and 0.1 mg/mL streptomycin. Cells were maintained at 37 °C in a humidified atmosphere with 5% CO_2_ and 95% air. The culture medium was refreshed every 2–3 days [21].

Cell Viability Assay

Extracts from two date varieties exhibiting the highest antioxidant capacities were selected for cell culture testing. After evaporating ethanol, the dry extracts were dissolved in dimethyl sulfoxide (DMSO) to achieve a concentration of 100 mg/mL. This stock solution was serially diluted with culture medium to final DMSO concentrations of 30 μg/mL, 50 μg/mL, 100 μg/mL, 190 μg/mL, 250 μg/mL, 380 μg/mL, 500 μg/mL, 750 μg/mL, and 1000 μg/mL, ensuring a final DMSO concentration equal to or lower than 1% (*v*/*v*).

Cell viability was assessed using the MTT assay adapted from previous works [22,23]. Caco-2 cells were seeded into 96-well plates at a density of 3 × 10^4^ cells per well and incubated for 24 h at 37 °C until reaching 80% confluence. Subsequently, the medium was replaced with prepared extract dilutions, and cells were incubated for an additional 24 h. Following treatment, the extract solutions were removed, and fresh medium containing MTT reagent (0.5 mg/mL) was added to each well. The plates were incubated in the dark for 1 h at 37 °C to allow for formazan crystal formation. Then, the medium with MTT was removed, and the resulting formazan crystals were dissolved in DMSO. Finally, absorbance was measured at 540 nm. A 1% DMSO solution (*v*/*v*) served as a solvent control. All experiments were performed in triplicate, and results are expressed as mean ± SD of three independent experiments.

Cell viability was calculated using Equation (4).

(4) $$\%Cell\;viability=\frac{Mean\;absorbance\;of\;test\;wells}{Mean\;absorbance\;of\;control\;wells}\cdot 100$$

### 2.7. Statistical Analysis

All measurements were conducted in triplicate, and results are presented as mean ± standard deviation (SD). Statistical analyses were performed using GraphPad Prism 7 software (GraphPad Software, Inc., San Diego, CA, USA). One-way Analysis of Variance (ANOVA) was employed to assess differences among samples, followed by Tukey’s post hoc test to identify specific group differences. A significance level of *p* < 0.05 was considered statistically significant. This approach ensures a robust evaluation of the data, accounting for variability and facilitating accurate interpretation of the findings.

## 3. Results and Discussion

### 3.1. Computer Vision System Analysis

**Figure 1:**
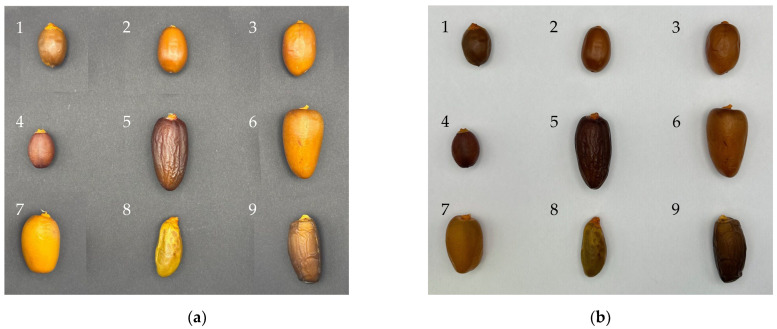
(**a**) Black background and (**b**) white background pictures of nine selected local date cultivars.

To standardize the visual analysis of the date samples, two sets of images were captured: one with a black background (Figure 1a) and another with a white background Figure 1b. The white background images were used to generate a segmentation mask, allowing for precise identification and isolation of each fruit by eliminating the influence of shadows and background noise. This mask was then applied to the corresponding black background images, which offered better contrast for visualizing the natural color of the dates. The color parameters (L*, a*, b*) were calculated from the segmented fruits in the black background images to ensure accurate and consistent color measurements under controlled lighting conditions.

**Figure 2:**
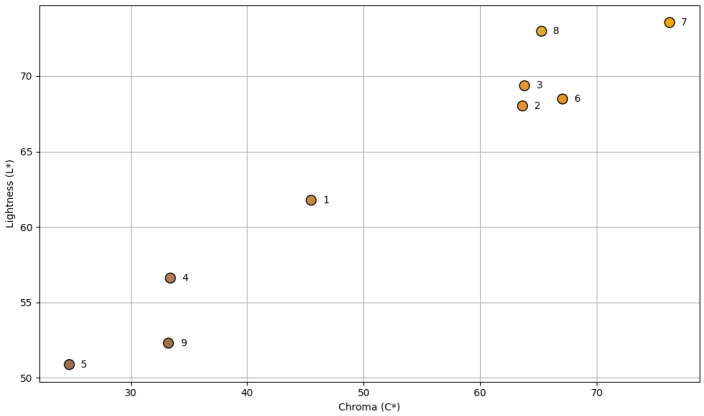
The relationship between chroma (C*) and lightness (L*) of fresh date samples of nine selected local date cultivars, with hue angle (h*) represented as a color gradient. Each point corresponds to an individual sample.

Color analysis of the date samples revealed a wide variation in chromatic attributes among the fruits collected from the Elche palm grove. The samples exhibited lightness (L*) values ranging from 50.88 to 73.55, chroma (C*) values from 24.74 to 76.25, and hue angles (h*) between 50.95° and 84.34°. This diversity in color parameters was visualized through a scatter plot (Figure 2) where chroma was plotted against lightness, and hue angle was encoded both as a color gradient and as a secondary y-axis. The distribution pattern suggested distinct chromatic identities among the samples, despite their common cultivation conditions.

The observed variation is closely tied to the intrinsic morphological diversity of the palms in the Elche palm grove. Unlike commercial orchards, where specific varieties (Medjoul and Confitera) are clonally propagated, the Elche palm grove is characterized by a high degree of genetic and phenotypic heterogeneity. This is due to its natural pollination strategy, whereby male and female flowers are left to pollinate freely rather than being artificially controlled. As a result, each palm tree acts as a genetically unique individual, producing fruits with potentially distinct appearances, colors, and sizes.

Despite this genetic diversity, date palms in the Elche palm grove are cultivated under heterogeneous agricultural conditions, ranging from irrigated plots to fallow lands and areas with spontaneous vegetation. These differences in management practices coexist with the intrinsic genetic variability of the trees. The fact that such a wide range of color values emerged across samples (D1–D9) suggests that the observed variation reflects not only environmental or management factors but also the biological uniqueness of each palm tree [24].

Color variation, particularly in terms of hue angle and chroma, is not only visually evident but also has implications for identifying morphological types and differentiating potential landraces or natural cultivars within the palm grove. Samples with lower lightness and chroma values tended to exhibit deeper brown or reddish tones, while those with higher values appeared more golden or amber-like. Color traits may reflect differences in ripening stage, sugar content, or surface texture, as reported in previous studies [8].

In conclusion, the colorimetric data not only provided a reliable method for sample characterization but also served as a proxy to explore the underlying phenotypic and genetic variability within the Elche palm grove. These results support the idea that even in non-standardized varietal contexts, objective image-based analysis can be a powerful tool for documenting and understanding agrobiodiversity in traditional farming systems.

### 3.2. Mineral and Heavy Metal Composition

Table 1 shows the content of macronutrients of nine selected local date cultivars. While the concentrations of carbohydrates, proteins, phenolic compounds, carotenoids, and antioxidant capacity were expressed per mL of hydroethanolic extract, reflecting their availability in the soluble fraction, the mineral and heavy metal content was determined directly in the freeze-dried date flour matrix and is therefore expressed per g or kg of dry weight. This methodological difference stems from the nature of the analyses: extractable compounds were assessed in solution, while elemental analysis required total acid digestion of the solid sample. Moreover, analyzing minerals and heavy metals in the freeze-dried flour rather than in the extracts ensured complete recovery of both soluble and insoluble fractions, providing a more accurate estimation of total elemental content and potential safety concerns. This approach complements the subsequent extract-based analyses, which focus on the bioavailable and functional components of the date matrix.

A more comprehensive interpretation of the results can be achieved by referring to the Recommended Dietary Allowance (RDA). Regardless of the date variety, potassium was the macronutrient present in the highest concentration, which is consistent with the findings of other researchers [8,25]. Comparing these values with the information presented by U.S. Food and Drug Administration, which recommends a daily potassium intake of 4700 mg for an adult, it can be considered that dates are a rich source of this macroelement. The cultivar with the highest potassium content (22.1 ± 0.30 mg/g) was D4. Except for the D6 cultivar, all tested samples exhibited potassium content higher than that reported for the commercially important Medjool variety cultivated in Mexico, for which a value of 8.52 mg/g has been documented by Salomón-Torres et al. [25]. These findings highlight the potential differences due to sample selection, cultivation conditions and geographical origin of the biological material. Maintaining an adequate supply of potassium is crucial due to the health benefits associated with its proper level in the body. Numerous studies have shown a correlation between insufficient potassium intake and an increased risk of hypertension. Despite cardiovascular health, potassium contributes to the proper functioning of muscles and transmission of nerve signals [26]. Magnesium is another macronutrient that plays a critical role in physiological processes. It acts as a cofactor in more than 300 enzymatic reactions, including energy generation and nerve and muscle function. Low magnesium levels have been linked to different diseases such as Alzheimer’s disease, migraine headaches, osteoporosis, and cardiovascular problems [27]. Average magnesium content in the nine tested date varieties was 75 mg per 100 g, which fulfills 17.85% of the daily recommended intake (420 mg); thus, dates can be considered a good source of this macronutrient. However, the magnesium content in all nine date cultivars was lower in comparison to the 1.43 mg/g reported for the Medjool variety originating from Mexico [25]. Calcium and phosphorus are vital for proper mineralization and, therefore, the optimal functioning of the skeletal system [28]. Moreover, calcium is a crucial regulator in nerve transmission and muscle contraction processes [29]. Phosphorus is relevant for DNA and RNA synthesis and plays an essential role in energy metabolism, as it is a component of ATP [30]. The average calcium and phosphorus concentrations per 100 g of tested date cultivars provide over 7.5% of the daily recommended intakes of these macronutrients. According to the results from Table 1, D8 was found to be the cultivar with the highest concentration of two out of four tested macronutrients (Mg and P). Although the variety with the highest potassium content was D4, D8 was also proven to be a good source of this macronutrient (13.0 ± 0.4 mg/g). As for calcium, the greatest concentration was found in cultivar D1, whereas D8 showed the second highest value (1.560 ± 0.07 mg/g) within the analyzed samples.

In accordance with Table 2, where the content of micronutrients in tested date cultivars is shown, the concentration of iron varied from 6.29 ± 0.13 (D5) to 15.32 ± 0.17 mg/kg (D8). The average content of 0.964 mg per 100 g of dates accounts for approximately 12% of the daily recommended iron intake, indicating that dates can be considered a good source of this essential microelement. Iron, as a fundamental component of hemoglobin, significantly contributes to oxygen transport throughout the body. Additionally, this micronutrient is a constituent of myoglobin, which ensures a proper supply of oxygen for muscle function. Beyond its role in oxygen transport, iron is essential for DNA synthesis and energy production. Iron deficiency may lead to anemia, a condition marked by inadequate oxygen distribution and reduced overall physical function [31]. Copper serves as a cofactor for various essential enzymes, highlighting its critical physiological significance as a micronutrient. Alongside iron, copper is vital for cellular energy production, contributing to ATP synthesis. Additionally, copper is critical for the absorption and transport of iron, thus enabling the synthesis of hemoglobin and efficient oxygen delivery within the body [32]. The average copper concentration in 100 g of dates was 0.352 mg, representing more than one-third of the daily recommended intake, which allows dates to be perceived as a rich source of this microelement. Zinc is a trace element that forms various enzymes and transcription factors, making it responsible for many processes occurring in the body, such as gene expression regulation or cell growth. Zinc also supports the proper functioning of the immune system and cognitive function [33]. Selenium is a fundamental component of a group of proteins known as selenoproteins, which are vital for numerous biological processes in the body. It serves as a cofactor of glutathione peroxidase, an enzyme responsible for protecting cells against oxidative damage. Comparable to zinc, selenium is also involved in the immune system response, improving the defense against infections by stimulating the activity of immune cells [34]. The average concentration of selenium and zinc in 100 g of dates corresponded, respectively, to 7.27 and 6.08% of the daily recommended intakes. The highest levels of these elements were detected in cultivar D8, which also exhibited the greatest concentrations of the other two analyzed microelements. Compared to our findings, the Medjool cultivar from Mexico exhibited different micronutrient composition with reduced iron (3.1 mg/kg) and zinc (2.6 mg/kg) content and elevated copper concentration (10.3 mg/kg) [25]. However, the micronutrient analysis of different date varieties cultivated in Morocco revealed higher zinc values, more comparable to those observed in our study (2.1–5.7 mg/kg). Moreover, some cultivars also showed similarly lower copper concentrations (3.4–4.9 mg/kg) [35].

The determination of heavy metal content is crucial to ensure the safety of date consumption. The risk of accumulation of heavy metals may be influenced by various factors such as environmental conditions, industrial pollutants and agricultural practices. The use of pesticides and contaminated water may introduce these metals into the plant. The issue is exacerbated in urban areas, where vehicle exhaust contributes to the release of heavy metals [36].

Among the analyzed metals (Table 3), lead exhibited the highest average concentrations, ranging from 16.5 ± 1.0 µg/kg in D1 to 113.0 ± 2.0 µg/kg in D8. Cadmium levels were considerably lower, ranging from below 1 µg/kg in D2, D6, D7, and D9 to 15.4 ± 0.8 µg/kg in D8. According to Commission Regulation (EU) 2023/915, the maximum permissible limits for lead and cadmium in fruits are 0.1 mg/kg (100 µg/kg) and 0.05 mg/kg (50 µg/kg), respectively. Although the concentration of lead in D8 slightly exceeded its regulatory threshold when expressed on a dry matter basis, the regulatory limits refer to fresh weight. Considering the dilution effect of water in fresh fruits, the actual concentrations would be lower, confirming that all tested samples remain within safe limits for both lead and cadmium. Nevertheless, the relatively higher values observed in D8 may indicate the influence of environmental or cultivation-related factors that require further monitoring.

Arsenic concentration varied between 17.7 ± 1.2 µg/kg in D5 and 45.8 ± 1.8 µg/kg in D1, with all values falling within recognized safety margins. Although Commission Regulation (EU) 2023/915 does not specify a maximum allowable limit for arsenic in fruits, the detected levels remain below the threshold suggested by international guidelines, such as those of the World Health Organization. As far as mercury is concerned, its presence in agricultural products is not common. All nine varieties exhibited the concentration of this heavy metal below 15 µg/kg.

**Table 3:** Heavy metal composition in nine selected local date cultivars (µg/kg dry mass). Results are expressed as mean ± SD of three independent replicates. Different letters in each column indicate statistically significant differences between samples (*p* < 0.05).

Cultivars	As (µg/kg Dry Mass)	Cd (µg/kg Dry Mass)	Hg (µg/kg Dry Mass)	Pb (µg/kg Dry Mass)
D1	45.80 ± 1.80 ^a^	2.00 ± 0.40 ^b^	<15.00	16.50 ± 1.00 ^e^
D2	37.50 ± 1.20 ^c^	<1.00 ^d^	<15.00	33.90 ± 1.60 ^c^
D3	29.40 ± 1.90 ^d^	1.46 ± 0.03 ^b^	<15.00	43.40 ± 1.80 ^b^
D4	29.20 ± 1.50 ^d^	1.29 ± 0.02 ^b^	<15.00	31.90 ± 1.10 ^c^
D5	17.70 ± 1.20 ^f^	1.64 ± 0.04 ^b^	<15.00	17.50 ± 0.60 ^e^
D6	19.70 ± 0.60 ^f^	<1.00 ^d^	<15.00	25.40 ± 1.10 ^d^
D7	25.00 ± 1.00 ^e^	<1.00 ^d^	<15.00	41.00 ± 1.40 ^b^
D8	20.40 ± 1.30 ^f^	15.40 ± 0.80 ^a^	<15.00	113.00 ± 2.00 ^a^
D9	41.90 ± 0.50 ^b^	<1.00 ^d^	<15.00	27.60 ± 0.90 ^d^

### 3.3. Carbohydrate and Protein Content

Following the assessment of mineral composition in the freeze-dried flour, the next analyses focused on the hydroethanolic extracts obtained through the green maceration process described above. These extracts represent the soluble and bioaccessible fraction of the date matrix, encompassing carbohydrates, proteins, phenolics, carotenoids, and antioxidant compounds. Beyond serving as analytical samples, they were conceived as functional, food-grade fractions, aligning with the study’s aim of developing sustainable ingredients from non-commercial date fruits. The following sections present their compositional and bioactive characterization.

**Figure 3:**
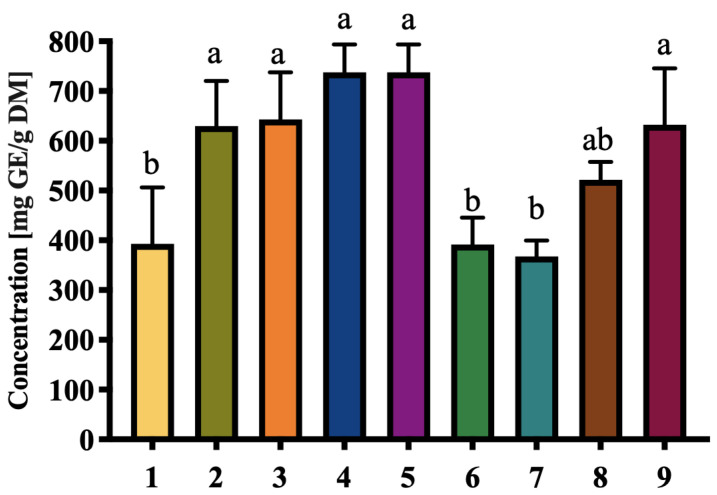
Total carbohydrate content of extracts of nine selected local date cultivars (mg glucose equivalent/g dry mass). Results are expressed as mean ± SD of three independent replicates. Different letters indicate statistically significant differences between samples (*p* < 0.05).

The carbohydrate content of the nine local palm date cultivars (Figure 3) varied significantly, ranging from 368.00 ± 31.91 mg GE/g DM in D7 to 737.70 ± 55.79 mg GE/g DM in D4 and D5. These results confirm that dates are an excellent source of carbohydrates, predominantly in the form of natural sugars such as glucose and fructose. The high carbohydrate concentration in D4 and D5 suggests their suitability for energy-rich food products, particularly for athletes or individuals requiring quick energy sources. This highest sugar level was comparable to that reported for the Sukari variety from Saudi Arabia (736.00 ± 63.00 mg/g DM) [37], suggesting that some local cultivars from Elche palm grove may reach carbohydrate concentrations comparable to commercial varieties.

Carbohydrates also play a role in product texture and stability, making date flour a valuable ingredient in baked goods and energy bars. Compared to other natural carbohydrate sources, dates offer the advantage of being minimally processed while retaining high nutritional density, as evidenced by studies highlighting their role in glycemic control [38,39].

**Figure 4:**
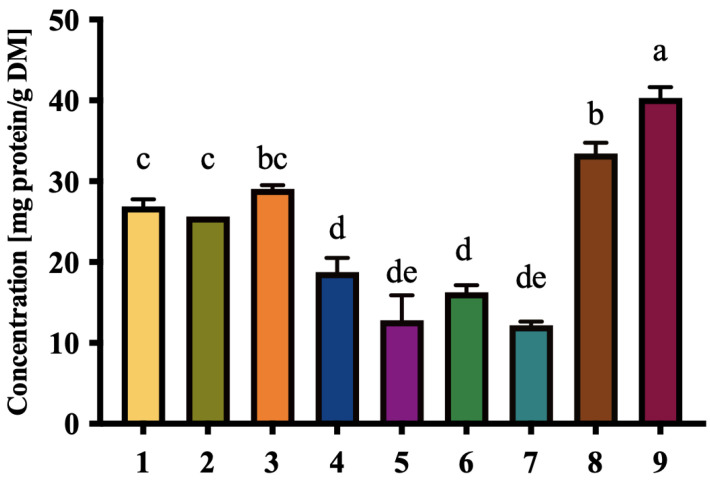
Total protein content of extracts of nine selected local date cultivars (mg protein/g dry mass). Results are expressed as mean ± SD of three independent replicates. Different letters indicate statistically significant differences between samples (*p* < 0.05).

Protein content across the cultivars was lower than carbohydrates but varied significantly, with D9 showing the highest levels (40.31 ± 1.33 mg/g DM) (Figure 4). Additionally, the cultivar with the highest concentration exhibited more protein than the five Saudi date varieties reported by Jaouhari et al., which ranged between 25.8 and 32.4 mg/g DM [37]. Despite a relatively modest protein contribution, dates can complement other protein sources in food formulations. This is particularly relevant in plant-based diets, where proteins derived from fruits can serve as supplementary sources of essential amino acids. Notably, the protein in dates was found to contain more than 20 different amino acids, including several that are not commonly found in popular fruits such as bananas, apples, and oranges [9]. Additionally, the presence of bioactive peptides, often overlooked in date proteins, warrants further investigation into their potential health benefits.

### 3.4. Phenolic and Carotenoid Content

**Figure 5:**
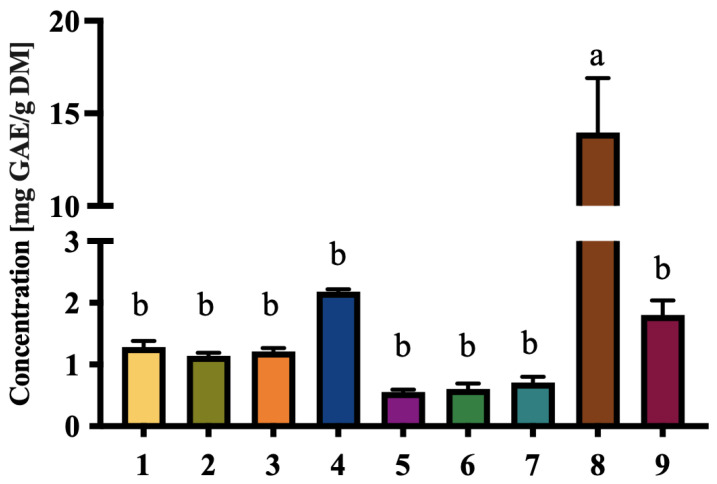
Total phenolic compound (TPC) values (mg gallic acid equivalents/g dry mass) of extracts of nine selected local date cultivars. Results are expressed as mean ± SD of three independent replicates. Different letters indicate statistically significant differences between samples (*p* < 0.05).

The phenolic content varied significantly among the nine cultivars (Figure 5), with D8 demonstrating the highest concentration (13.98 ± 2.93 mg GAE/g DM). Phenolics are known to provide a range of health benefits, including anti-inflammatory, anti-carcinogenic, and cardiovascular-protective effects [40]. The TPC of the other tested local cultivars was generally comparable to values reported for Saudi date palm varieties, such as Ajwa (1.14 mg GAE/g DM) and Anbarah (0.81 mg GAE/g DM) [41], as well as to Moroccan date cultivars, which ranged from 1.19 to 4.10 mg GAE/g DM [42].

The elevated levels in D8 suggest its potential as a functional food ingredient with high antioxidant properties. Additionally, phenolic compounds may contribute to flavor enhancement, as they are associated with bitterness and astringency, which could be leveraged in gourmet or artisanal food products.

The color of fruits partially reflects the presence of pigments such as carotenoids, which are responsible for the yellow, orange, and red hues in plant-based products [43]. Literature reports indicate that high color saturation often correlates with elevated levels of these natural pigments, as demonstrated by Takagi et al. [44]. In our study, cultivars 2 and 8, which showed the highest carotenoid content (36.44 ± 1.55 μg/g DM and 31.27 ± 1.01 μg/g DM, respectively), exhibited similarly high chroma values (C* > 60). Moreover, D5 and D9, which presented the lowest carotenoid content (1.14 ± 0.09 μg/g DM and 1.91 ± 0.03 μg/g DM, respectively), displayed low chroma values (C* < 35).

**Figure 6:**
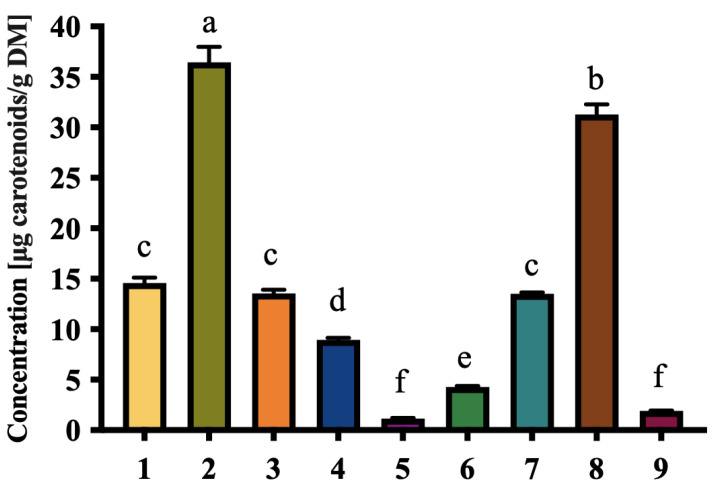
Carotenoid content of nine local cultivars of palm date extracts (μg carotenoids/g dry mass). Results are expressed as mean ± SD of three independent replicates. Different letters indicate statistically significant differences between samples (*p* < 0.05).

The carotenoid levels (Figure 6) observed in cultivars 2 and 8 exceeded the upper range previously reported for Omani dates (9.2–29.1 µg/g DM) [45]. Additionally, although earlier studies have shown that carotenoid concentrations tend to decline with fruit ripening, these two cultivars, collected at the Tamar stage, still exhibited comparatively high values, suggesting that certain local cultivars may retain higher pigment levels even at advanced maturity [46].

As carotenoids are known for their antioxidant properties and health benefits—particularly in vision and suppression of oxidative stress—the cultivars with elevated levels underline the potential of these fruits to serve as natural colorants and nutraceutical ingredients [47]. Carotenoid-rich date varieties may offer potential for use in formulations targeting eye health or antioxidant enhancement, but their practical implementation requires prior evaluation of bioaccessibility and stability. [48]. Furthermore, carotenoids’ vibrant yellow-to-orange hues could enhance the aesthetic appeal of functional foods, especially in beverages or dairy alternatives.

### 3.5. Total Antioxidant Capacity

Antioxidant capacity was evaluated using two complementary methods: Trolox Equivalent Antioxidant Capacity (TEAC) and Oxygen Radical Absorbance Capacity (ORAC). These assays assess the ability of date extracts to neutralize free radicals, providing a comprehensive understanding of their antioxidant potential. Among the tested cultivars, D8 exhibited the highest antioxidant activity with TEAC and ORAC values of 43.47 ± 4.15 μM TE/g DM and 74.40 ± 5.83 μM TE/g DM, respectively. In contrast, D5 recorded the lowest values, with TEAC at 0.68 ± 0.03 μM TE/g DM and ORAC at 18.53 ± 0.40 μM TE/g DM.

Higher TPC values tended to be associated with higher TAC, highlighting the potential contribution of phenolic compounds to the antioxidant properties of date extracts. Polyphenols are known to donate hydrogen atoms to free radicals, stabilizing them and preventing oxidative damage to biomolecules such as lipids, proteins, and DNA. These findings align with previous studies that have established a direct relationship between polyphenolic content and antioxidant activity [49,50].

In addition to their health benefits, antioxidants play a crucial role in food technology. They inhibit lipid peroxidation, which is a primary cause of rancidity in fat-rich foods. Future studies could investigate the antioxidant potential of date extracts in lipid-based systems to assess their suitability as clean-label alternatives to synthetic antioxidants in food preservation, such as butylated hydroxytoluene (BHT) and butylated hydroxyanisole (BHA) [51].

Overall, the strong antioxidant potential of date extracts, especially from D8, supports their dual role as both health-promoting ingredients and functional additives. Future research could focus on the stability of these antioxidant properties during food processing and storage, as well as their bioavailability in human systems. Understanding these factors would further solidify the position of date flour as an ingredient in functional and fortified foods.

### 3.6. Cell Viability

The chemical analysis of nine date cultivars identified two with the highest antioxidant potential, highlighting them as promising candidates for further investigation. The application of cell line models enables the in vitro evaluation of the biological activity of bioactive compounds contained in dates, including their potential protective effect on cellular functions. The impact of two date palm extracts with the highest antioxidant potential—D8 and D4—on Caco-2 cells was assessed over the concentration range from 30 μg/mL up to 1000 μg/mL.

**Figure 7:**
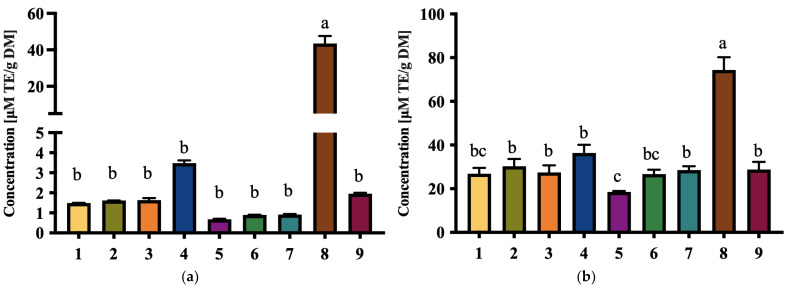
(**a**) Trolox equivalent antioxidant capacity (TEAC) and (**b**) Oxygen Radical Absorbance Capacity (ORAC) (mM Trolox equivalents/g dry mass) of extracts of nine selected local date cultivars. Results are expressed as mean ± SD of three independent replicates. Different letters indicate statistically significant differences between samples (*p* < 0.05).

**Figure 8:**
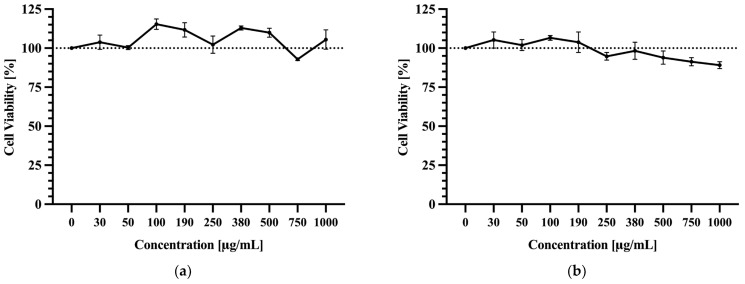
Effect of D8 (**a**) and D4 (**b**) extract on Caco-2 cell viability, at concentrations ranging from 30 up to 1000 µg/mL, after 24 h of exposure and measured by MTT assay. The results are presented as mean ± SD of three independent experiments. The dashed line at 100% represents the viability of control cells treated with 1% DMSO and serves as the reference baseline for all treatments.

As presented in Figure 7, D8 was found to be the cultivar with exceptionally high antioxidant capacity. Its extract demonstrated a dose-dependent effect on Caco-2 cell viability. Low concentrations enhanced viability compared to control conditions, particularly at a concentration of 100 μg/mL, where cell viability peaked at 115%. It is worth noting that across all tested concentrations, viability remained close to or above 100%. At the higher concentration (0.75 mg/mL), cell viability was reduced slightly below 100% but consistently exceeded 90% (Figure 8a).

The second extract tested for cell viability assessment was obtained from cultivar D4. Although its antioxidant capacity value was significantly lower than that of D8, it was also included to broaden the spectrum of results. As shown in Figure 8b, at low concentrations (from 30 to 190 μg/mL), D4 extract, similar to the other cultivar, promoted cell viability compared to the control. The highest value was observed at the concentration of 100 μg/mL, reaching 106%. Higher extract doses tended to decrease the cell viability sooner compared to cultivar D8, with viability falling below 100% at concentrations exceeding 190 μg/mL. At the highest tested concentration (1000 μg/mL), viability was reduced to 90%.

Importantly, no statistically significant differences (*p* ≤ 0.05) in cell viability were observed between treated and control samples for both extracts. These results demonstrate that tested concentrations of both date palm extracts did not exhibit a significant cytotoxic effect on Caco-2 cells under the studied conditions. Similar observations were reported in the research conducted by Barrera-Chamorro et al. [52], which tested the effect of phenolic extracts obtained from olive by-products on Caco-2 cell viability and their immunomodulatory properties. The authors demonstrated no cytotoxicity at concentrations between 10 and 200 μg/mL of the tested extracts. Notably, in this study, there was also observed a tendency for cell viability to decrease with increasing extract concentration, but these changes, as in our case, were not statistically significant.

Further research that includes longer exposure times, additional cell models, and genotoxicity assays is needed to comprehensively assess the safety of date palm extracts for food applications. At the same time, their high phenolic content suggests promise for future investigation exploring potential anti-inflammatory and immunomodulatory effects.

Taken together, these findings suggest that the hydroethanolic extracts obtained through a green maceration process may combine chemical richness with biological safety, supporting further investigation into their potential as natural functional ingredients in sustainable food formulations.

## 4. Conclusions

This exploratory study provides evidence that Ilicitano freeze-dried date flour and its green hydroethanolic extracts may exhibit relevant nutritional and bioactive properties, suggesting their potential as functional food ingredients. The analyses revealed substantial nutritional value, particularly in carbohydrates, minerals, and phenolic compounds, with the D8 extract showing comparatively higher antioxidant activity and micronutrient levels. The absence of cytotoxic effects in Caco-2 cells suggests preliminary safety at the tested concentrations, although the elevated lead levels observed in D8 highlight the need for continued monitoring and additional safety evaluations.

The combined assessment of flour and extract matrices provided an initial overview of the compositional and functional features of Elche dates, obtained through a sustainable, food-grade extraction process. The computer vision approach additionally supported the visual documentation of color and morphological variability, illustrating the link between phenotypic diversity and compositional differences within the palm grove.

Overall, these findings represent an exploratory but valuable contribution toward the characterization and potential valorization of non-commercial Elche dates. As the study was limited to one harvest season and a single geographic region, further research should include additional trees and seasons, assess bioaccessibility and extraction yield, and expand safety testing to other biological models to fully validate these results and their applicability in functional food development.

## Figures and Tables

**Table 1:** Macronutrient content in nine selected local date cultivars (mg/g dry mass). Results are expressed as mean ± SD of three independent replicates. Different letters in each column indicate statistically significant differences between samples (*p* < 0.05).

Cultivars	Mg (mg/g Dry Mass)	P (mg/g Dry Mass)	K (mg/g Dry Mass)	Ca (mg/g Dry Mass)
D1	1.026 ± 0.008 ^b^	0.605 ± 0.007 ^b^	10.820 ± 0.160 ^c^	1.940 ± 0.040 ^a^
D2	0.698 ± 0.011 ^e^	0.490 ± 0.007 ^d^	11.530 ± 0.180 ^c^	1.030 ± 0.020 ^d^
D3	0.972 ± 0.009 ^c^	0.594 ± 0.005 ^b^	13.250 ± 0.110 ^b^	1.320 ± 0.030 ^c^
D4	0.374 ± 0.006 ^i^	0.500 ± 0.010 ^d^	22.100 ± 0.300 ^a^	0.453 ± 0.013 ^f^
D5	0.608 ± 0.007 ^f^	0.505 ± 0.004 ^d^	9.190 ± 0.070 ^e^	0.710 ± 0.020 ^e^
D6	0.565 ± 0.008 ^g^	0.549 ± 0.008 ^c^	8.800 ± 0.200 ^e^	0.451 ± 0.016 ^f^
D7	0.476 ± 0.005 ^h^	0.488 ± 0.017 ^d^	13.400 ± 0.200 ^b^	0.524 ± 0.012 ^f^
D8	1.232 ± 0.012 ^a^	0.776 ± 0.007 ^a^	13.000 ± 0.400 ^b^	1.560 ± 0.070 ^b^
D9	0.810 ± 0.030 ^d^	0.480 ± 0.005 ^d^	10.000± 0.400 ^d^	0.750 ± 0.030 ^e^

**Table 2:** Micronutrient content in nine selected local date cultivars (mg/kg dry mass or µg/kg dry mass). Results are expressed as mean ± SD of three independent replicates. Different letters in each column indicate statistically significant differences between samples (*p* < 0.05).

Cultivars	Fe (mg/kg Dry Mass)	Zn (mg/kg Dry Mass)	Cu (mg/kg Dry Mass)	Se (µg/kg Dry Mass)
D1	9.54 ± 0.11 ^d^	8.00 ± 0.40 ^b^	2.97 ± 0.03 ^e^	48.00 ± 3.00 ^b^
D2	9.03 ± 0.05 ^de^	8.20 ± 0.30 ^b^	4.86 ± 0.04 ^b^	44.00 ± 3.00 ^b^
D3	11.41 ± 0.17 ^b^	8.40 ± 0.20 ^b^	2.58 ± 0.04 ^f^	43.10 ± 1.80 ^b^
D4	8.83 ± 0.09 ^e^	2.83 ± 0.08 ^f^	3.62 ± 0.04 ^c^	30.00 ± 2.00 ^cd^
D5	6.29 ± 0.13 ^g^	4.54 ± 0.10 ^e^	3.41 ± 0.02 ^d^	26.30 ± 1.20 ^d^
D6	9.35 ± 0.04 ^d^	5.93 ± 0.09 ^d^	3.39 ± 0.02 ^d^	40.00 ± 3.00 ^bc^
D7	7.11 ± 0.07 ^f^	6.70 ± 0.20 ^c^	2.23 ± 0.03 ^g^	22.90 ± 1.40 ^d^
D8	15.32 ± 0.17 ^a^	9.20 ± 0.30 ^a^	5.22 ± 0.05 ^a^	68.00 ± 4.00 ^a^
D9	9.94 ± 0.18 ^c^	6.40 ± 0.20 ^cd^	3.39 ± 0.06 ^d^	33.90 ± 1.60 ^c^

## Data Availability

The original contributions presented in this study are included in the article. Further inquiries can be directed to the corresponding author.
